# Incidence and predictors of chronic kidney disease in type-II diabetes mellitus patients attending at the Amhara region referral hospitals, Ethiopia: A follow-up study

**DOI:** 10.1371/journal.pone.0263138

**Published:** 2022-01-26

**Authors:** Medina Abdela Ahmed, Yohannes Mulu Ferede, Wubet Worku Takele

**Affiliations:** 1 Department of Medical Nursing, School of Nursing, College of Medicine and Health Sciences, University of Gondar, Gondar, Ethiopia; 2 Department of Community Health Nursing, School of Nursing, College of Medicine and Health Sciences, University of Gondar, Gondar, Ethiopia; Magna Graecia University of Catanzaro: Universita degli Studi Magna Graecia di Catanzaro, ITALY

## Abstract

**Background:**

Chronic kidney disease (CKD) is the severest form of kidney disease characterized by poor filtration. The magnitude of chronic kidney disease is trending upward in the last few years linked with the rapidly escalating cases of non-communicable chronic diseases, particularly diabetes mellitus. However, little is known about when this problem may occur, the incidence as well as predictors of chronic kidney disease among type-II diabetes mellitus patients. Thus, this study was conducted to determine the incidence, time to the occurrence, and predictors of chronic kidney disease in type-II diabetic patients attending the Amhara region referral hospitals, Ethiopia.

**Methods:**

A retrospective follow-up study was conducted involving 415 participants with type-II diabetes mellitus that enrolled in the chronic follow-up from 2012 to 2017. Multivariable shared Frailty Weibull (Gamma) survival model was employed considering the hospitals as a clustering variable. Model fitness was checked by both the Akaike information criteria (AIC) and log-likelihood. Factors having a p-value of ≤0.2 in the bi-variable analysis were considered to enter the multivariable model. Variables that had a p-value of <0.05 with its corresponding 95% confidence level were deemed to be significant predictors of chronic kidney disease.

**Results:**

The overall cumulative incidence of chronic kidney disease was 10.8% [95%; CI: 7.7–14.0%] with a median occurrence time of 5 years. The annual incidence rate was 193/10,000 [95%; CI: 144.28–258.78]. Having cardiovascular disease/s [AHR = 3.82; 95%CI: 1.4470–10.1023] and hypercholesterolemia [AHR = 3.31; 95% CI: 1.3323–8.2703] were predictors of chronic kidney disease.

**Conclusion:**

One out of every ten diabetic patients experienced chronic kidney disease. The median time to develop chronic kidney disease was five years. Hypercholesterolemia and cardiovascular diseases have escalated the hazard of developing CKD. Thus, health promotion and education of diabetic patients to optimize cholesterol levels and prevent cardiovascular disease is recommended to limit the occurrence of this life-threatening disease.

## Introduction

Diabetes mellitus (DM) is a metabolic disorder associated with either the failure of the pancreatic islet beta cells that produce insulin, or insulin resistant where the human body cannot uptake the available insulin effectively [1]. DM is alarmingly increasing and becoming one of the pressing public health problems among other non-communicable chronic diseases (NCD) globally [2]. The prevalence of DM was 8.8% among people between the age group of 20 and 79 years, which indicates almost 440 million people are affected by the problem. It is predicted that more than 550 million people will develop DM by the end of 2035 [3]. Different vascular and neural damages, including kidney disease, are attributable to DM that might pose danger in the renal capillaries and subsequently lead to the reduction of glomerular filtration rate (GFR) [4]. Once the kidney is damaged, it could not filter properly, and difficult to remove waste products that interfere with the normal physiological function of the body which progressively leads the body to shutdown [5].

CKD is a progressive loss of kidney function resulted from the vascular and neural complications of DM that incites several adults to death prematurely [4–7]. In the world, around 13.3 million people are affected by CKD yearly, of which 85% of the cases are from developing countries Approximately, 1.7 million annual deaths is ascribed to kidney disease [8]. The annual incidence of CKD among type-II diabetic patients ranges from 20-58/1000 [9–15]. Moreover, the time-to-occurrence of CKD (the time at which patients developed CKD since they diagnosed with type II DM) varies across different studies. The median occurrence time of CKD was around 3.8–12 years worldwide [9, 10, 16, 17]. In Africa, the incidence of CKD is estimated to be between 13.3–25% [18–21]. Particularly, in Sub Saharan Africa, the burden of CKD is much greater associated with additional risk factors like poverty, infections, low level of health literacy, and the high cost of medical fees for screening and treatment, that collectively aggravate the risk and progression of the problem with declined probability of survival [22, 23].

Ethiopia is one of the developing countries with a high burden of CKD due to the emerging of NCD associated with the swift changes in lifestyle [24]. About 10.4–19.1% of the population has exhibited CKD in the country [25–27]. The median time to develop CKD among type-II DM was also estimated to be 5.9 years [27]. Likewise, 39% of annual deaths in the country are owing to NCD, of which diabetes-associated kidney failure makes up 10 to 40 percent of all deaths [28]. CKD in type II DM patients is attributable to multifaceted factors such as dyslipidemia, overweight/obesity, co-morbidity (hypertension), cardiovascular diseases, and uncontrolled blood glucose levels [9, 10, 27, 29, 30].

Ethiopia has signed to achieve the Sustainable Development Goal (SDG) from 2016 to 2030 that includes reducing premature death from NCD by a third [31]. However, little is known related to the incidence, time to occurrence of CKD, and its predictors, particularly among type II DM patients.

Therefore, the aim of this study was tripled (i) to estimating the time-to-occurrence of CKD; (ii) determining the incidence of CKD; and (iii) identifying factors predicting CKD among T2DM patients attending in the Amhara region referral hospitals. Understanding the aforementioned research problems will help decision-makers to cut the morbidity and mortality rates associated with CKD. Furthermore, clinicians may also use this evidence and strengthen the care provided to diabetic patients.

## Methods and materials

### Study design and period

A retrospective follow-up study was conducted by reviewing medical recordings of type-II DM adult patients who have had follow up from the 1^st^ of May 2012 to the 1^st^ of May 2017 in the Amhara region referral hospitals, Ethiopia. The data were collected during April 1^st^ and May 1^st^, 2021.

### Study setting

Northwest Amhara regional state is situated to the north of Addis Ababa, the capital of Ethiopia. In the region, there are 6 referral hospitals, namely the University of Gondar comprehensive specialized hospital, Felege Hiwot referral hospital, Tibeb Gione referral hospital, Debere Markos referral hospital, Debre Berhan referral hospital, Debre Tabor referral hospital. These referral hospitals have a system to provide care for chronically ill patients having different forms of illnesses, including patients with DM. Currently; roughly 12,300 clients with type-II DM visit these hospitals to receive regular follow-up care.

### Study population and sample size determination

Patients aged 30 years and older who were diagnosed with type-II DM who enrolled in the chronic follow-up care and receiving regular follow-up care in the Amhara region referral hospitals from 2012 to 2017 were included.

The sample size was determined by the sample size determination formula for time to an event $\;n=\frac{{\left(\frac{Z\alpha }{2}+Z\beta \right)}^{2\;}}{b2P1P2d}$. Where ’n’ is the required sample size; $\frac{“Z\alpha ”}{2}$ is the critical value of standard normal distribution variable at 95% significant level, “*Zβ*” is the critical value of the standard normal distributed variable at 20% *β*; ‘‘b”is lan of hazard ratio (lan HR); "d" is the prevalence of an event CKD) = 0.15; and Hazard ration of 0.11 [27].

$$\text{ne}=\underline{{\left(1.96+0.84\right)}^{2}}$$

$$\text{lan}{\left(0.11\right)}^{2}\left(0.116*0.484\right)0.15$$

ne=191

n=191*2=382

and adding 10% non-response and the final sample size was 420.

### Sampling technique and procedure

Of the five referral hospitals, four hospitals (i.e the University of Gondar comprehensive specialized hospital, Felege Hiwot referral hospital, Debere Markos referral hospital, and Debre tabor referral hospital) were selected randomly.

Proportional sample allocation was done for each referral hospital based on the monthly number of DM patients who were on follow-up. Finally, the required number of participants was selected by simple random sampling technique using the list of diabetic patients enrolled between the 1^st^ of May 2012 and the 1^st^ of May 2017 as a sampling frame. Then, using a computer-generating random sample technique, 420 participants were chosen.

### Data collection tool and procedure

The data were gathered from the medical recordings of patients enrolled in the chronic follow care from the 1^st^ of May 2012 to the 1^st^ of May 2017. A data extraction checklist was used to abstract the required data. The socio-demographic characteristics, the presence of co-morbidity, treatment modality, blood glucose status, and lipid profiles of the clients were extracted. The data were gathered by 4 BSc nurses supervised by two MSc nurses.

### Quality assurance

To maintain the quality of the data, a one-day training focusing on the data extraction system, data collection tools, and objectives of the study was given to data collectors and supervisors. The quality of the data was attempted to assure by taking 5% (21 patient charts) of the total participants to pretest at Tibebe Gion referral hospital before starting the data collection. After analyzing the pretest data, some variables were added, and the language ambiguity was cleared. Close follow-up and supervision were carried out during the data collection period jointly by the principal investigator and the supervisors. The necessary feedbacks have been forwarded to the data collectors daily. The collected data were reviewed and checked for its completeness before data entry. Misclassification bias was attempted to minimize by using uniform ascertainment criteria for those clinical variables having more than one diagnostic criteria. Moreover, the study was reported using the Strengthening the Reporting of Observational Studies in Epidemiology (STROBE) checklist for follow-up [32].

### Operational definitions

**CKD**: eGFR of < 60 ml/ min/1.73 m^2^ for at least three months [27].
**Time-to-CKD**- the time between the diagnoses of type-II DM to the detection of CKD.
**Event**: CKD.
**Censored**: explained by patients who had not been diagnosed with CKD until the end of the study, or died, or lost to follow-up, or transferred out.
**HTN**: a systolic blood pressure ≥140 mmHg or diastolic blood pressure ≥90mmHg or reported the regular use of the anti-hypertensive drug [33].
**Cardiovascular disease**: confirmed any type of cardiovascular disease/s.
**The complication of DM**: any acute or chronic complication of DM which is registered on the chart.
**Elevated Low Density Lipoprotein** (LDL) ≥120 mg/dl is abnormal [34].
**Low level of High-Density Lipoprotein** (HDL): < 40 mg/dl and <50mg/dl for male and female, respectively, otherwise declared to be normal [35].
**High Triglyceride** (TGL) ≥ 150 mg/dl [36].
**Elevated total cholesterol level** ≥ 200 mg /dl is abnormal [34].

### Data processing and analysis

Data entered, cleaned, and coded using Epi Info version 7 and exported to STATA version 14 for further analysis. Continuous variables were described in terms of mean and median along with the appropriate measure of dispersions.

The incidence rate of CKD was also calculated for the entire cohort by dividing the total number of incident cases of CKD by the total person-years of follow-up. To estimate the median survival time and compare it across groups of key characteristics, the survival curves and Kaplan-Meier were used.

The proportional hazard assumption test was checked graphically, by using Schoenfeld residual test and Cox-Snell residual test, and the assumption was not violated. Variables having a p-value ≤ of 0.2 were entered into the models. Considering the clustering effect by the hospital (the heterogeneity of incidence of CKD across hospitals), a multivariate shared frailty model with Weibull distribution and Gamma frailty term was applied. This model has been chosen after comparing it with Cox and other parametric models with different distributions and frailty terms. Model fitness was checked by both the Akaike information criteria (AIC) and log-likelihood. The model was selected considering the AIC and log-likelihood estimates. The presence of multicollinearity was checked by using the variance inflation factor and correlation coefficient. Variables having a p-value < 0.05 in the final model were considered to be significantly associated with the CKD. The risk estimate was expressed using an adjusted hazard ratio with its 95% CI.

### Ethical consideration

Ethical approval was obtained from the institutional review board (IRB) of the University of Gondar (Ref.No:-S/N164/7/2013). A permission letter to conduct the study was received from the medical director’s office of the included Hospitals. The data clerks of the hospital were adequately informed about the purpose, method, and anticipated benefits of the study by the data collector. Since the source of data was secondary (chart review), a consent was not applicable and waiver has been permitted by the IRB.

## Results

### Socio-demographic characteristics

A total of 415 type-II diabetic patients with a response of 98.8% were included. Over half (52%) of the participants were female. Just below three-fourths (71.6%) were married. Their mean age was 56.13 (SD 10.2) years. Well over half (59%) of the study participants were aged between 51 and 70 years. Further, just under three-fourths (72.5%) of them were urban residents (Table 1).

**Table 1:** Socio-demographic characteristics of type-II diabetics patients attending the Amhara region referral hospitals, Ethiopia 2021(n = 415).

Variables	Frequency	Percent (%)
**Age**		
30–64	329	79.3
≥65	86	20.7
**Marital status**		
Single	15	3.6
Married	297	71.6
separated	7	1.7
Divorced	28	6.7
Widowed	68	16.4
**Ethnicity**		
Amhara	389	93.7
Tigire	23	5.6
Others	3	0.7
**Religion**		
Orthodox	297	71.6
Muslim	106	25.5
Protestant	12	2.9

### History of medical illness

Close to two-thirds (61%) of the participants developed hypertension, while 20% of them had cardiovascular diseases. A small proportion (5.1%) of participants developed retinopathy. Approximately, a fourth (23.9%) of the study participants developed acute and chronic complications of DM. More than a quarter (29.6%) of them have been taking aspirin (Table 2).

**Table 2:** History of medical illness among type-II DM patients attending in the Amhara region referral hospitals, Ethiopia 2021 (n = 415).

Variables	Number	Percent (%)
**Type of cardiovascular disease(83)**		
CHF	29	34.9
Ischemic heart disease	22	26.5
Peripheral artery disease	15	18.1
Stroke	17	20.5
**Other Co-morbid diseases**		
Yes	53	12.8
No	362	87.2
**List of co-morbid illnesses (53)**		
RVI	14	26.4
Asthma	9	17
BPH	5	9.4
TB	7	13.2
PUD	5	9.4
Breast cancer	4	7.6
Epilepsy	2	3.8
Goaut arteritis	6	11.3
Toxic multi nodular goiter	1	1.9
**List of complications of DM(99)**		
Acute complication		
DKA	46	46.5
HHNS	15	15.2
**Chronic complications**		
Neuropathy	13	13.1
Nephropathy	17	17.1
Foot ulcer	8	8.1
**Rx given for type II DM patients**		
Oral hypoglycemic drug	279	67.2
Oral hypoglycemic drug + insulin	136	32.8

### Incidence and medina time of CKD

The participants were followed up for a minimum of 4 and a maximum of 9 years with 5 years of median time. Throughout the follow-up, 45 diabetic patients had developed CKD with 2329 person-years. The incidence rate of CKD was 0.0193(193/10,000persons/year) [95%CI; 144.26–258.78]. The cumulative incidence of CKD was 10.8% [95%; CI: 7.7–14.0]. The cumulative incidence rate of CKD in patients attending the University of Gondar specialized comprehensive referral hospital, Felege Hiwot referral hospital, Debre Tabor general referral hospital, and Debre Markos referral hospital was 10.6%, 12.7%, 10.7%, and 8.5%, respectively (Table 3).

**Table 3:** Incidence of CKD by referral hospitals in western Amhara referral hospitals (n = 415).

Name of the referral hospital	Number of CKD occurred	Person year	Incidence rate/10,000/year	[95% Conf. Interval]
**UOGCSRH**	16	798	200	(122.8–327.3)
**FHRH**	15	718	208	(125.9–346.5)
**DTRH**	6	284	211	(94.9–470.2)
**DMRH**	8	529	151	(75.6–302.30)

UOGCSRH: University of Gondar Comprehensive Specialized Referral Hospital.

FHCRH: Felege Hiwot Referral Hospital.

DTRH: Debre Tabor Referral Hospital;

DMRH: Debre Markos Referral Hospital.

### Kaplan- Meier recovery estimate and smoothed hazard estimate

**Fig 1:**
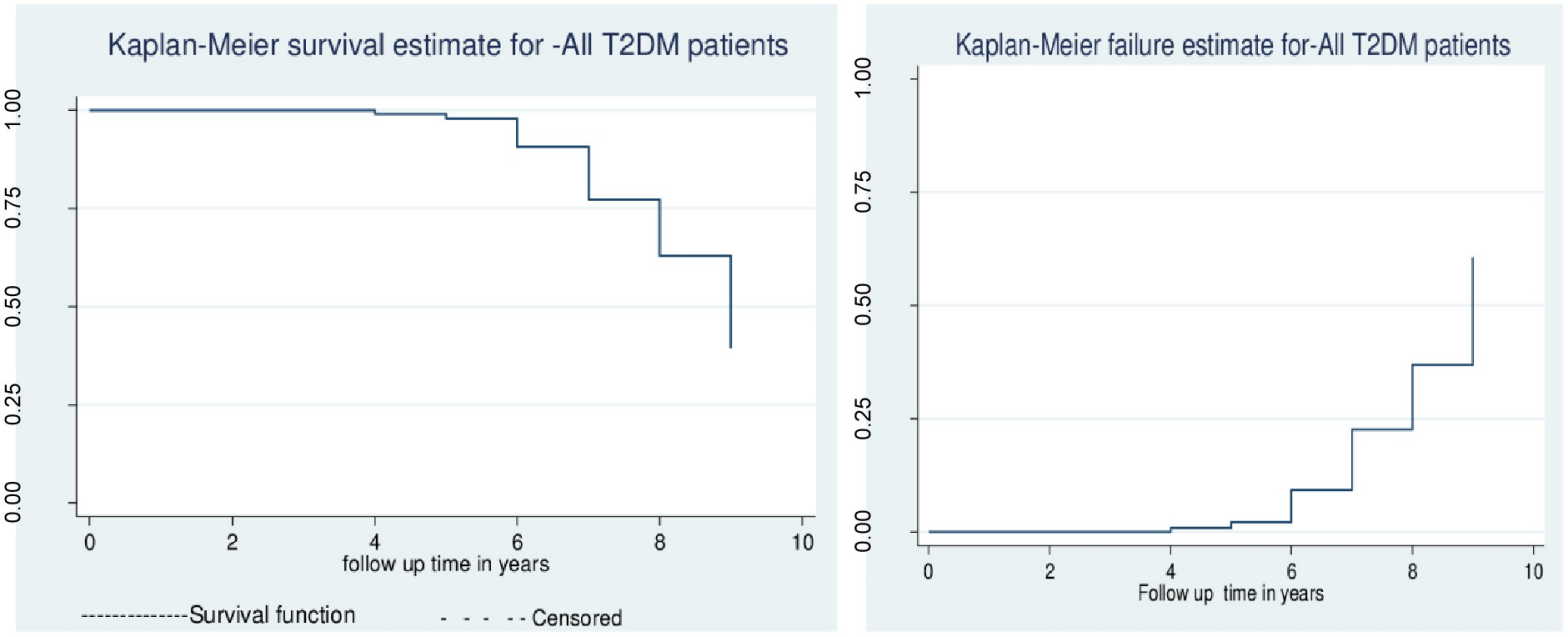
Kaplan- Meier recovery estimate and smoothed hazard estimate of CKD among type two diabetic patients in western Amhara referral hospitals (n = 415).

**The median survival time of the study participants was 9 years. The graph showed that the probability of T2DM patients who survived during the follow-up time throughout each time interval (0, 2, 4, 6, 8, and 9 years)**. As time goes on, T2DM patients are less likely to survive from CKD. In the 4^th^ year, the survival time of diabetic patients’ is 100% while the time goes to 8 years, the survival rate reaches 70% (Fig 1).

### Kaplan- Meier graphs for predictors

Patients whose serum total cholesterol level greater than or equal to 200mg/dl vis-à-vis less than 200mg/dl, their survival time was equal for the first 5 years. However, at the 8^th^ year of the follow-up, the survival time of participants whose serum cholesterol level was greater than equal to 200mg/dl and less than 200mg/dl was at 60% and 75%, respectively (p = 0.010). The median survival time of patients whose serum cholesterol level was greater than equal to 200mg/dl was eight years, while it’s 9 years among those whose serum cholesterol level was less than 200mg/dl.

**Fig 2:**
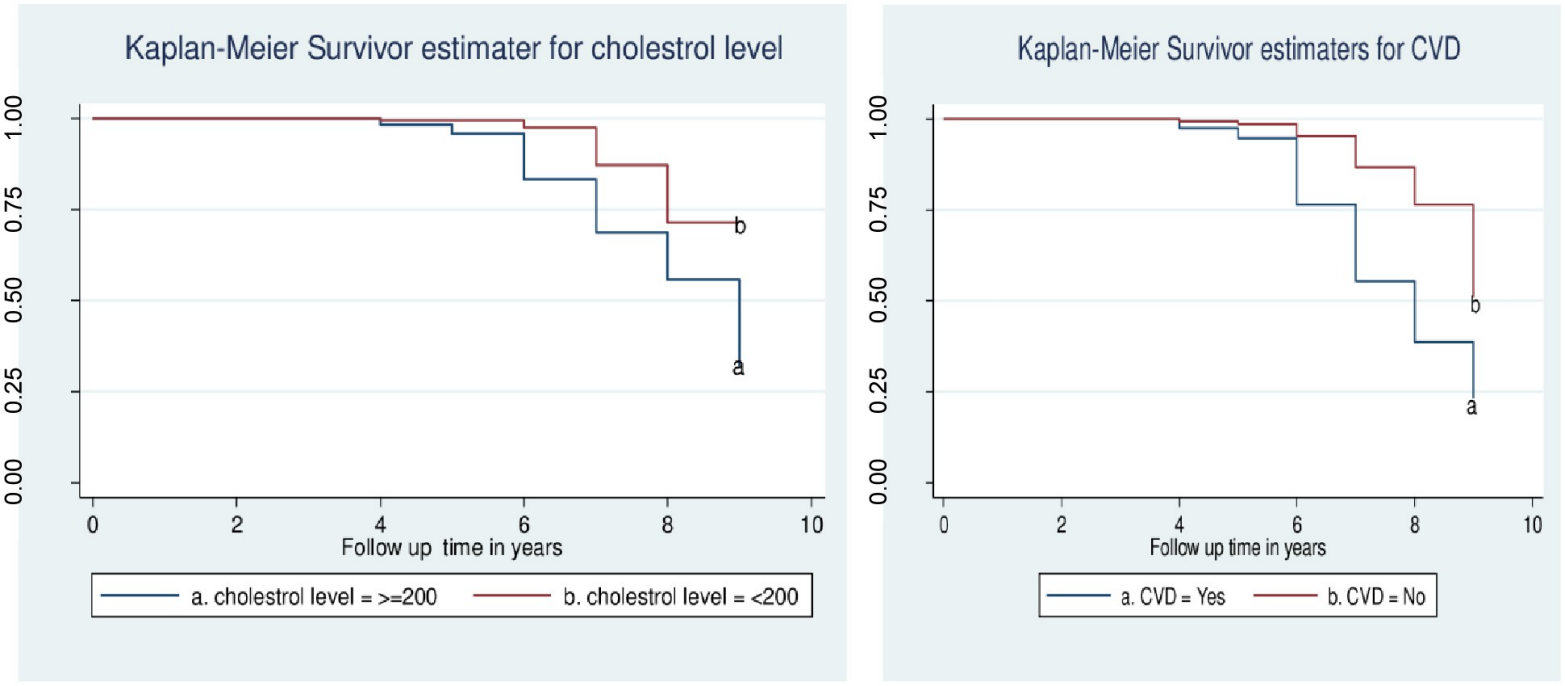
Kaplan- Meier graphs for predictor variables of CKD among type two diabetic patients attending in the Amhara region referral hospitals (n = 415).

The probability of survival from CKD among study subjects who developed cardiovascular diseases was lower than their counterparts. At the 8^th^ year of the follow-up, the survival probability of participants who developed cardiovascular diseases was around 40%, while participants who didn’t develop cardiovascular diseases was 80% (Fig 2).

### Predictors of CKD among type II diabetic patients

Age, sex, the presence of other complications of DM, cardiovascular disease, history of ASA intake, fasting blood glucose level, LDL, and total cholesterols were predictors at p-value ≤ 0.2 and transferred to the final model.

In the multivariable shared frailty Weibull (Gamma) (considering hospital as a clustering effect) model, however, only cardiovascular disease and total cholesterols were significant predictors of CKD. Accordingly, among diabetic patients who developed cardiovascular disease, the hazard of experiencing CKD was more than three times higher than study subjects who didn’t develop cardiovascular disease [AHR = 3.82;95%CI:1.4470–10.1023]. Similarly, the hazard of CKD among patients who had hypercholesterolemia was 3 times higher compared to their counterparts [AHR = 3.3168; 95%CI: 1.3323–8.2703] (Table 4).

**Table 4:** Bivariable and multivariable Shard Frailty Weibull (Gamma) survival model analysis for predictors of CKD among type II diabetics patients attending western Amhara referral hospitals, Ethiopia 2021(n = 415).

Variables	Status		CHR (95% CI)	AHR (95% CI)	P-value
	CKD	Censored			
**Age**					
30–64	28	301	0.592(0.323–1.085)	0.92(0.438–1.937)	0.829
≥65	17	69	1	1	1
**Sex**					
Female	15	201	0.491(0.263–0.919)	0.60(0.313–1.167)	0.134
Male	30	169	1	1	1
**Confirmed Hx of Cardiovascular diseases**					
Yes	27	56	3.17(1.729–5.813)	3.82(1.447–10.102)**	0.007
No	18	314	1	1	1
**Complication of type II DM**					
Yes	25	74	2.31(1.271–4.213)	2.00(0.984–4.078)	0.055
No	20	296	1	1	1
**Hx of ASA Rx**					
Yes	26	97	1.722(0.944–3.143)	0.44 (0.169–1.158)	0.097
No	19	273	1	1	1
**FBG level**					
≥250mg/dl	35	173	2.58(1.269–5.269)	1.26(0.573–2.770)	0.564
<250 mg/dl	10	197	1	1	1
**LDL level**					
≥120 mg/dl	31	151	1.245(0.637–2.435)	0.791(0.381–1.639)	0.529
<120 mg/dl	12	144	1	1	1
**Total cholesterol level**					
≥200 mg/dl	36	145	3.165(1.467–6.831)	3.31(1.332–8.270)*	0.010
<200 mg/dl	9	192	1	1	1

**Note**: superscript ‘*’ indicates p-value less than 0.05, ‘**’ indicates p-value less than 0.01.

## Discussion

CKD is the severest stage of renal problem that could be irreversible and leads to death. This disorder is commonly observed in patients with NCD primarily DM. Thus, determining the incidence, median occurrence time, and its predictors would have a paramount contribution to take appropriate and timely measures to ensure the survival of the victims. Therefore, the study looked at the incidence, the median time to the occurrence of CKD, and its predictors among type-II DM patients attending referral hospitals in the Amhara region.

Throughout the follow-up, out of 415 diabetic patients, 45 developed CKD with a cumulative incidence rate of 10.8% [95%; CI: 7.7–14.0]. The total person-time was 2329 person-years with an incidence density of 0.0193(193/10,000persons-year). The finding indicated that the observed problem is a public health important issue that deserves due attention. Taking into account the upward trend of DM and the poor self–care practice among victims in the country, the problem may be escalated unless a grave measure is not in place [37, 38]. The cumulative incidence of CKD in this study was in line with findings from studies done in Italy (13.4%) [13], China (12.7%) [14], and Spain (10.23%) [15]. However, it’s considerably lower than studies done in elsewhere Ethiopia (14.25%) [27] and Sweden (20%) [39]. The observed discrepancy might be because all the study participants involved in the previous study based in Ethiopia were urban dwellers in that their lifestyle might distort the metabolic system that would further lead to renal impairment. In this study, however, 27% of study participants were from rural areas. Variation in the applied methods between Sweden’s and this study could be the reason for the observed incidence difference. Similarly, the outcome ascertainment used in the former study was measured using albumin, unlike this study which used eGFR. Moreover, it’s known that Sweden is a well-developed country in that the variation in the patients’ health-seeking behavior, the quality of professionals working in these settings, and the availability of advanced diagnostics equipment may help clients to screen ahead [40].

The median occurrence time of CKD among type-II DM patients was 5 years, which is congruent with a study done in North America (4.4 to 4.7years) [9]. However, it’s deemed to be shorter than the findings of the studies done in the capital of Ethiopia (Addis Ababa) (5.9 years) [27], the UK (12years) [10], and Australia (5.7years) [16]. This could be ascribed to the quality of the service, the patients’ awareness about complications of DM, health-seeking behavior of patients’, their residency (all urban), and self-care practice were better among participants involved in the current study [41]. The formers studies conducted in Addis Ababa, the UK, and Australia recruited participants residing in civilized cities such that their awareness and health-seeking behavior is expected to be better, which could enable them to apply better self-care practice [42]. The median time in this study is higher than the population-based study done in Canada (3.8years) [17] and China (3.3 years) [43]. The difference might be due to method variability, for instance, Canada’s study is a community-based that might have included participants who may not have regular follow-up time in health facilities which may accelerate the occurrence of complications notably CKD.

The study revealed that patients with hypercholesterolemia have a higher incidence of CKD than those who have normal cholesterol levels. This finding is in agreement with studies done in Spain [15], Australia [44], Taiwan [45], and China [46]. This might be associated with the effect of high cholesterol levels causing, cholesterol plaque that can block the blood flow to the kidneys through the renal arteries, compromising the kidney’s function and increasing the likelihood of CKD [47]. Another mechanism could be high cholesterol level increases the reabsorption of phospholipids by tubular epithelial cells that lead to dyslipidemia; this phenomenon could raise the level of LDL which aggravates the formation of proinflammatory cytokines that induces glomerulosclerosis [48, 49]. Moreover, hypercholesterolemia results in hypertension, stroke, and other related medical complications apart from CKD, and thus, regular monitoring of cholesterol levels and taking appropriate measures are largely suggested especially for type II DM patients.

Likewise, cardiovascular diseases are significant predictors of CKD among type II DM patients. The hazards of CKD among diabetic patients who develop at least one cardiovascular disease are three times higher than patients who didn’t have the disease. The finding is supported by studies done in Spain [15] and the UK [10].

The association of cardiovascular disease and CKD might be when the heart and/or supporting vasculatures is/are started to function abnormally, the heart might not be capable of pumping sufficient blood out. Thus, the heart may become too full of blood that causes pressure in the main vein connected to the kidneys, which progressively leads to a blockage and reduced supply of oxygen-rich blood supply to the kidneys that again provide a route to develop CKD [50]. This association was also supported by different findings that demonstrated, lower cardiac output and decreased effective circulating blood volume result in baroreceptor stimulation, increased sympathetic nervous activity, and renin secretion that prompt increased sodium reabsorption and glomerular mesangial cells constriction that would narrow the filtration area of the glomerulus [51]. In general, the finding of this study implies the need for preventing cardiovascular disease to curve the risk of CKD.

The multicenter nature of the study could allow reflecting the regional burden of CKD and making generalizations. However, there are undeniable shortcomings of the study. This study is a retrospective follow-up that was based on the analysis of routinely collected data such that certain types of data such as laboratory results were missed.

## Conclusion

One out of every ten diabetic patients experienced CKD. The median time to develop CKD is five years. Further, hypercholesterolemia and cardiovascular diseases have accelerated the hazard of developing CKD. Hence, participants with DM are recommended to prevent cardiovascular diseases and maintain their cholesterol levels. Clinicians are also suggested to educate clients aiming at preventing cardiovascular disease and high cholesterol levels as well as to provide more emphasis for these patients for early screening and management. Moreover, researchers in the field are suggested to conduct a prognostic study to identify predictors using risk scores so that clinicians will uptake the evidence and prioritize patients during treatment.

## Supporting information

S1 Data (DTA)

## Abbreviations

CKD: Chronic Kidney Diseases
AIC: Akakie Information Criteria
DM: Diabetes Mellitus
eGFR: estimated Glomerular Filtration Rate
HDL: High-Density Lipoprotein
HTN: Hypertension
LDL: Low-Density Lipoprotein
NCD: Non-Communicable Disease
SDG: Sustainable Development Goal
T2DM: Type 2 Diabetes Mellitus
TGL: Triglyceride
WHO: World Health Organization
